# eDNA Metabarcoding Reveals Microbial Community Composition in Tropical Mangrove Forests in Makassar, Indonesia

**DOI:** 10.1002/ece3.72740

**Published:** 2026-01-11

**Authors:** Siti Halimah Larekeng, Mohammad Basyuni, Andi Aznan Aznawi, Irmawati Irmawati, Iswanto Iswanto, Muhammad Saldy, Alfian Mubaraq, Bejo Slamet, Elham Sumarga, Virni Budi Arifanti, Hayssam M. Ali

**Affiliations:** ^1^ KARST Bioprospecting and Society, Institute of Research and Community Service Hasanuddin University Makassar Indonesia; ^2^ Research Collaboration Center for KARST Microbes, National Research and Innovation Agency‐Institute of Research and Community Service Hasanuddin University Makassar Indonesia; ^3^ Faculty of Forestry Hasanuddin University Makassar Indonesia; ^4^ Center of Excellence for Mangrove Universitas Sumatera Utara Medan Indonesia; ^5^ Department of Forestry, Faculty of Forestry Universitas Sumatera Utara Medan Indonesia; ^6^ Doctoral Program of Agriculture Science, Faculty of Agriculture Universitas Sumatera Utara Medan Indonesia; ^7^ Faculty of Marine Science and Fisheries Hasanuddin University Makassar Indonesia; ^8^ School of Life Sciences and Technology Institut Teknologi Bandung Bandung Indonesia; ^9^ Research Center for Ecology National Research and Innovation Agency Cibinong Indonesia; ^10^ Department of Botany and Microbiology, College of Science King Saud University Riyadh Saudi Arabia

**Keywords:** bioinformatics analysis, conservation strategy, e‐DNA metabarcoding, mangrove Lantebung, microbial species

## Abstract

Mangrove ecosystems are critical coastal habitats that support diverse microbial communities essential for nutrient cycling, organic matter decomposition, and ecosystem stability. Despite their ecological importance, microbial diversity in tropical mangroves remains poorly characterized, particularly in Indonesia. This study employed environmental DNA (eDNA) metabarcoding via 18S rRNA markers to assess microbial species diversity in the waters of the Lantebung Mangrove Forest, Makassar, Indonesia. Water samples were collected from two locations and analyzed through high‐throughput sequencing and bioinformatic processing via QIIME 2. A total of 103 microbial species were identified across five kingdoms: Protista (48.5%), Chromista (28.0%), Animalia (12.6%), Bacteria (7.8%), and Fungi (2.9%). The dominant taxa included photosynthetic protists and chromists, along with functionally important bacterial and fungal species. Species richness and relative abundance differed among the sampling sites, reflecting environmental gradients such as light availability, salinity, and nutrient flow. Notably, Station P1 (more exposed) presented greater species richness, whereas Station P2 (within the mangrove canopy) presented greater individual abundance. These findings highlight the effectiveness of 18S rRNA‐based metabarcoding in capturing microbial diversity and offer valuable baseline data for future ecological monitoring and conservation strategies in tropical mangrove ecosystems.

## Graphical abstract

This study used environmental DNA (eDNA) metabarcoding with 18S rRNA markers to assess microbial diversity in the Lantebung Mangrove Forest, Makassar, Indonesia. It identified 103 microbial species across five kingdoms, revealing higher species richness in open‐water areas (P1) and greater individual abundance under mangrove canopy (P2). The findings provide essential baseline data for biodiversity monitoring and conservation in tropical mangrove ecosystems.

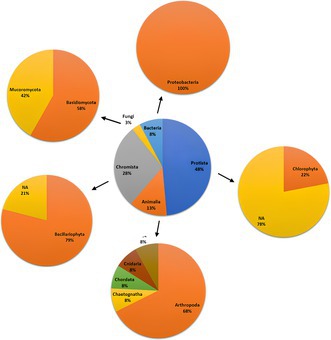

## 1 Introduction

The mangrove area in Makassar city covers 133,000 ha, accounting for approximately 2% of the total mangrove area in Indonesia. Despite its relatively small size, this ecosystem holds significant importance for the local community. Its limited extent also underscores the need to prioritize its protection and long‐term preservation (Riska 2022). South Sulawesi Province has high potential for coastal resources and small islands, starting from coral reefs, seagrasses, mangroves, beaches, rivers, and estuaries. The potential of these natural resources, such as marine tourism on small islands, has been developed by the city government. In 2001, the mangrove area was only approximately 50.30 ha, and in 2015, it experienced an additional area of 58.53 ha or an increase of approximately 16% (Bando et al. 2017). Currently, the Makassar Lantebung mangrove area has reached 12 ha. This occurred because various conservation and mangrove planting activities in the northern coastal area of Makassar city have been carried out by various parties (Riska 2022).

Each mangrove species, including microbial species, has different adaptations to various biotic and abiotic stresses (Thomsen 2012). Biotic factors such as plant species composition and root exudates and abiotic conditions such as salinity, temperature, pH, and turbidity play crucial roles in shaping microbial community structure. Understanding mangrove microbial diversity is essential for revealing the ecological functions these microbes perform, such as nutrient cycling, organic matter decomposition, and the ability to support plant health. The identification of mangrove microbial diversity can be performed via various methods, including biochemical approaches and molecular techniques. Recent developments have used environmental DNA metabarcoding technology to evaluate the biodiversity of an ecosystem (Basyuni et al. 2021).

Biodiversity analysis methods can be carried out with a metabarcoding approach, which combines DNA taxonomy and DNA sequence technology to maximize species‐level identification of tissue remnants that are not detected by conventional methods (Harsms‐Tuohy et al. 2016). In metabarcoding, DNA samples are isolated in large quantities, amplified with marker genes, and sequenced, followed by comparisons obtained from databases, thus allowing for correct community assessment and streamlining time. Pavan‐Kumar et al. (2015) reported that DNA metabarcoding is used for multispecies identification via total DNA, which is usually isolated from environmental samples or from whole‐sample organisms. Multiple‐species identification techniques were originally applied to microbial communities or often called metagenomics, but they are now beginning to be applied to eukaryotic organisms such as fungi, invertebrates, plants, and vertebrates (Ushio et al. 2017). Therefore, DNA metabarcoding is the latest method for large‐scale DNA analysis (Laramie et al. 2015).

Mangrove ecosystems harbor highly complex and dynamic microbial communities that play critical roles in ecosystem functioning, including nutrient cycling, decomposition of organic matter, and maintaining overall ecosystem health (Alongi 2014). Recent advances have highlighted that traditional cultivation‐based methods greatly underestimate the true microbial diversity of eukaryotes in these environments. High‐speed sequencing techniques, such as metabarcoding based on 18S rRNA gene markers, enable more comprehensive and accurate profiling of microbial communities by detecting both abundant and rare taxa (Lallias et al. 2015). The application of the environmental DNA (e‐DNA) metabarcoding method aims to assess biodiversity where samples are taken from the environment through sediment, water, and air, including whole cells, extracellular DNA, and potentially whole species (Takahara 2013). e‐DNA can be derived from the skin, mucus, saliva, sperm, secretions, eggs, feces, urine, blood, roots, leaves, fruit, pollen, and decaying bodies of larger organisms, while microorganisms can be obtained as a whole. Environmental DNA metabarcoding results in differences in species composition. The development of variables to evaluate mangrove ecosystem function by metabarcoding environmental DNA is a very important endeavor (Miya et al. 2015).

Information on species diversity through the metabarcoding approach in the water area of the mangrove forest ecosystem in Lantebung has not been reported. In Indonesia, mangrove microbial diversity studies using metabarcoding remain limited. Globally, several studies have employed eDNA metabarcoding to assess microbial diversity in mangrove and coastal ecosystems. For example, Liu et al. (2021) studied eukaryotic microbial diversity in the sediments of Hainan Island, China, whereas Ting et al. (2021) evaluated urban microbial eukaryotes in Malaysia. Furthermore, eukaryotic microbial communities associated with coral, sediment, and seawater in Vietnam have been investigated (Linh and Van Ngoc 2023). These studies demonstrate the utility of 18S rRNA markers in diverse aquatic systems. However, no similar comprehensive study has been conducted in the Lantebung mangrove area of Makassar. This research aims to address this gap by applying the 18S rRNA‐based metabarcoding approach to explore microbial community composition in this unique coastal ecosystem.

## 2 Materials and Methods

### 2.1 Study Area and Environmental Conditions

**FIGURE 1:**
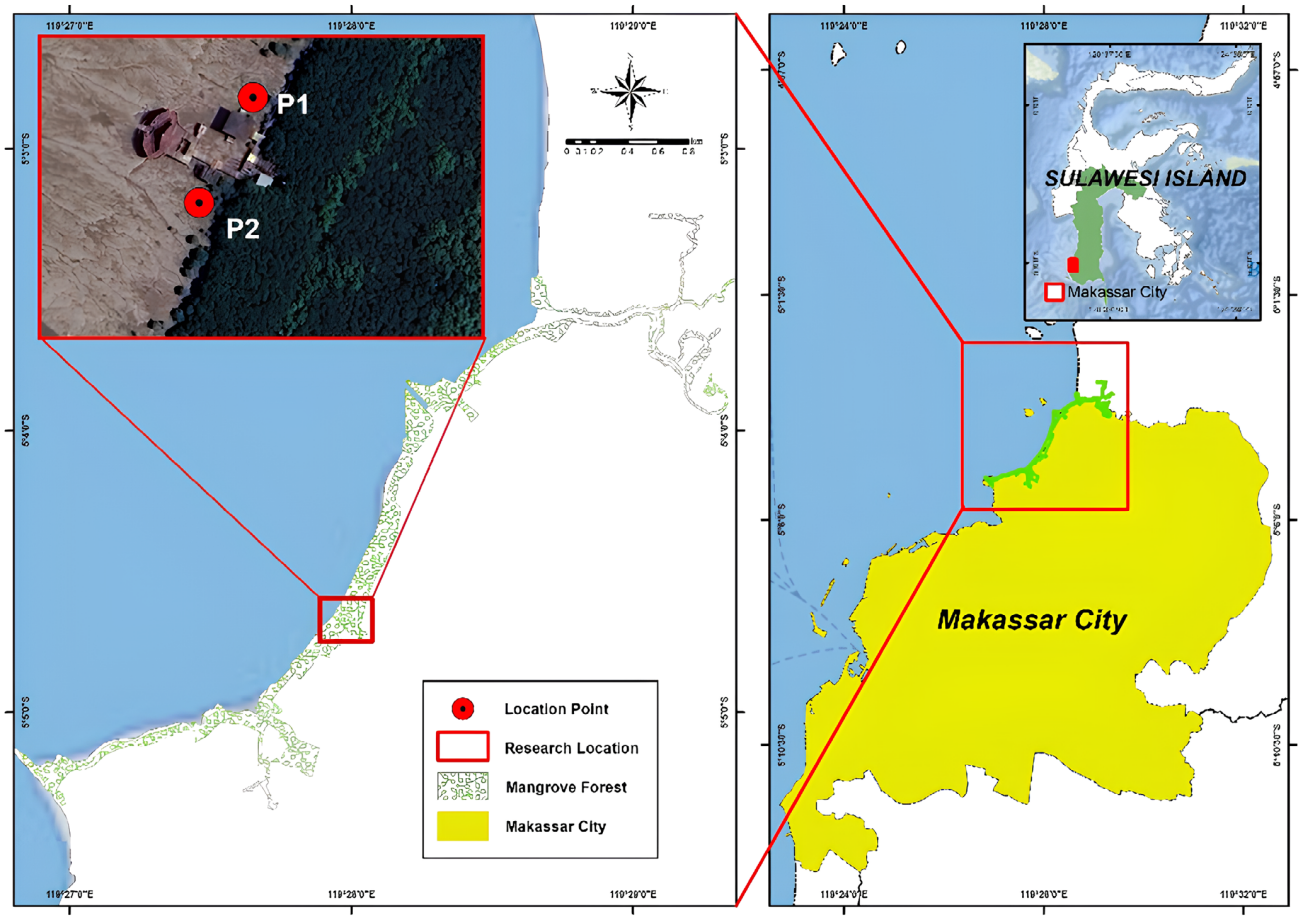
Study site of Lantebung Mangrove Forest.

The study site for this research is located in Lantebung Mangrove Forest, South Sulawesi, specifically at coordinates 5°04′37″ S–119°27′56″ E and 5°04′37″ S–119°27′56″ E (Figure 1). The research was conducted from December 2022 to May 2023. The samples used were taken from two points in the waters of the Makassar Lantebung Mangrove Forest, where these two points are symbolized by the codes P1 (to the north and toward the open sea) and P2 (to the south toward the mangrove plant) according to the cardinal direction, with the distance between the two stations being 250 m. Sampling began in the morning at 10:00 a.m., with sea conditions being high tide and quite choppy, resulting in turbid water conditions and a high sediment content. Sampling occurred during high tide under turbid water conditions. The environmental parameters at both the P1 and P2 stations were measured at the time of collection. These include water temperature (29.5°C–30.1°C), salinity (27–30 ppt), pH (7.5–8.1), and turbidity (high, due to wave action and sediment disturbance). These conditions influence microbial presence and diversity, especially in highly dynamic estuarine environments such as Lantebung.

### 2.2 Materials Sample

Water samples in the sediment section were collected at two different points (Madduppa et al. 2021; Zuhdi et al. 2021), and each sample bottle had a volume of 1 L. Water sampling was chosen to target planktonic and free‐living microbial eukaryotes, which are underrepresented in sediment studies. This approach complements prior sediment‐based mangrove microbiome studies (Liu et al. 2021). Water collection can be performed by directly submerging a sterile bottle into the water until the bottle is filled. The water sample is then filtered via a set of filtering equipment in the form of a peritaltic pump masterflex or water pump and filtered via 0.45 μm millipore paper in a set of bucher funnels. Filtering is carried out according to the manufacturer's protocol on the bucher funnel. The filter paper was cut into two pieces using scissors that had been sterilized with 70% bleach solution. The filter paper was inserted into a 2 mL cryotube that had been filled with ±1 mL of DNA shield liquid to preserve the sample.

### 2.3 DNA Extraction and Amplification

The filter paper eDNA from the samples was extracted via the Qiegen Blood and Tissue DNA Extraction Kit according to the manufacturer's instructions. The first PCR contained 12 μL of Kapa HotStart HiFi 2× ReadyMix DNA polymerase, 1 μL each of 10 nM primers (F and R), 8 μL of ddH2O, and 2 μL of the DNA template, and the PCR process used a PCR machine with the following conditions: (1) presaturation of mold DNA at 95°C for 5 min; (2) denaturation of mold DNA at 98°C for 30 s; (3) annealing at 65°C for 30 s; (4) primer extension at 72°C for 30 s; and (5) post extension at 72°C for 5 min with 35 cycles of stages (2–4). The PCR product quality was visualized via electrophoresis on 2% agarose GelRed (100 mL of TAE buffer and 2 g of agarose). A total of 3 μL aliquots of PCR products were then inserted into each agarose well with a 100 bp DNA ladder in one of the wells. The electrophoresis machine was run at 50 V for 60 min, and the results were visualized via fluorescent UV through the Alpha Imager Mini Gel Documentation System (Protein Simple Ltd., California, USA). The gel of the PCR bands was then purified via the AMPure XP Kit (Backman Coulter Inc.).

### 2.4 Amplification DNA (Library Preparation) and Next‐Generation Sequencing (NGS)

Library preparation and sequencing, which is also an extension of this method (Amaral‐Zettler et al. 2009), was performed in the previous step. All triplicate PCR products that passed electrophoresis quality control were subjected to a second PCR for indexing purposes. (a) The IDT dual index and Illumina sequencing adapter for the Illumina‐Nextera DNA Unique Dual Index, Set A (catalog number 20027213) (Illumina, San Diego, USA), were added to the target amplicons for PCR, and 12.5 μL of Kapa HotStart HiFi 2× ReadyMix DNA polymerase (Kapa Biosystems Ltd., London, UK) and 2 μL of PCR product were used. The primers used were Euk_1392F (5′‐GTACACACCGCCCGTC‐3′) and EukBR (5′‐TGATCCTTCTGCAGGTTCACCTAC‐3′) as previously reported (Hugerth et al. 2014). The PCR cycle consisted of an initial denaturation at 95°C (3 min), 9 cycles of 95°C for 30 s, 55°C for 30 s, and 72°C for 30 s, and a final extension at 72°C for 5 min. (b) PCR products were purified via AMPure XP. (c) DNA sequencing was performed on an Illumina iSeq100. All sequencing protocols were in accordance with the Illumina MiSeq 16S metagenomic sequencing library protocol. The primers used in this study are widely used for eukaryotic diversity studies because of their broad coverage and suitable length for Illumina sequencing platforms (Amaral‐Zettler et al. 2009; Liu et al. 2021).

### 2.5 Geographic Weighted Regression (GWR)

The identification of organisms was carried out via the taxonomic classification method, which groups sequences on the basis of their level of similarity to produce several different groups, known as operational taxonomic units (OTUs) (Wu et al. 2019). The sequences were analyzed via one of the bioinformatics pipelines, QIIME 2 (the Quantitative Insights Into Microbial Ecology 2 program, https://qiime2.org/), which is integrated and used to create microbiome data (Bolyen et al. 2019).

## 3 Results and Discussion

### 3.1 Effectiveness of 18S Primers in Detecting Microbial Species Diversity

After the data are processed and analyzed via bioinformatics software, information can be obtained about the number of haplotypes, unique haplotypes, and species variations found in mangroves (Chao et al. 2014), as shown in Table 1.

**TABLE 1:** Number of 18S sequences detected in the Makassar Lantebung mangrove area.

Sample ID	Input	Filtered	Denoised	Merged	Nonchimeric
P1	13,589	13,245	12,920	12,551	12,533
P2	15,388	15,069	14,774	14,502	14,492

In the early stages of bioinformatic analysis, raw data enter the pipeline so that the software reads how many DNA sequence reads are contained in each raw data (raw sequencing data). The raw data entered in the pipeline show that sample (P1) has 1799 data points, which is 13,589 fewer than sample (P2) 15,388. Sequencing data from Illumina's iSeq100 revealed 13,589 sequences at station (P1) and 15,388 sequences at station (P2). Station (P2) had more sequences than did station (P1). The sequences are then filtered. The input data results are then filtered on the basis of the length of the reference bp that matches the primer target. For sample P2, 15,069 data points were obtained, and 13,245 data points were obtained for sample P1. From this process, P1 data were reduced by 344 data points and (P2) 319 data points.

Data that have been filtered enter the denoising or filtering stage on the basis of DNA quality; only DNA with a quality score of 30 is used so that the P1 sample that passes to the denoised stage is 12,920, and the P2 sample has a quality score of 14,774. After denoising, the merged data, which combines the forward primer data and reverse primer data, were entered; in sample P1, 12,551 samples were merged.

While nonchimeric data are not chimeric data, these data are highly avoided in the analysis because they are generated not from DNA found in the field but from DNA data obtained from the DNA merger itself. In addition, station P1 has more data that are not used, namely 1056 sequences, whereas at station P2, there are 896 sequences.

### 3.2 Microbial Species Abundance in Lantebung via 18S rRNA Primers

In the analysis of species diversity in Makassar Lantebung Mangrove Forest waters, species variation was detected at each sample point in the mangrove waters. The results of the analysis revealed differences in species in the mangrove waters of the Makassar Lantebung Mangrove Forest. In addition, the results of the analysis revealed the variation in species in the mangrove population in the Makassar Lantebung Mangrove Forest.

One of the studies that used UEK primers was a study (Liu et al. 2021) on the diversity of eukaryotic microorganisms in marine sediments around Hainan Island, China. This study used UEK primers to amplify 18S rRNA fragments from eukaryotic microorganisms in sediment samples. The results revealed a high diversity of eukaryotic microorganisms in the marine sediments around Hainan Island.

Another study by Nguyen et al. (2022) also used UEK primers to identify the presence and diversity of eukaryotic organisms in water samples from river ecosystems in Vietnam. The results revealed the presence of many eukaryotic phyla, including *Alveolata*, *Stramenopiles*, and *Chloroplastida* in river water samples.

**FIGURE 2:**
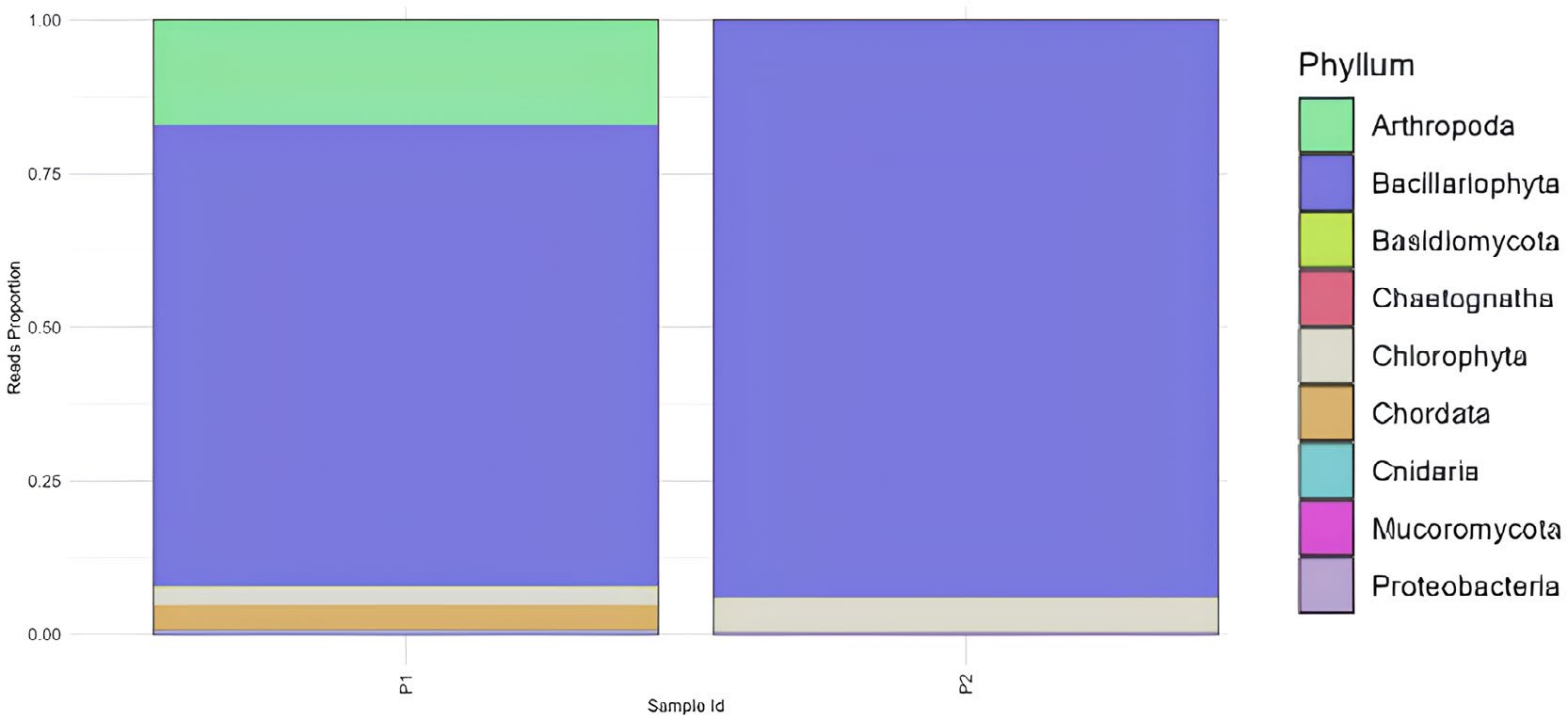
Taxa identified at the phylum level in samples P1 and P2.

In this study, the authors conducted DNA metabarcoding analysis to identify the species diversity of the Lantebung Mangrove Forest. The proportions of taxa obtained at the phylum level were as follows:

Figure 2 shows the diversity of the phylum‐level individuals in the Lantebung Mangrove Forest based on the 18S rRNA primers, and a comparison of the species diversity at the two stations (P1 and P2) is shown. Station P1 contained species from the phyla arthropods bacllariophyta, chlorophyta, chordata, proteobacteria, Basidiomycota, and cnidaria, whereas station P2 contained bacillariophyta, clorophyta, Mucoromycota, proteobacteria, arthropods, Basidiomycota, and Chaetognatha. In addition to the differences in species at the two stations, differences in the proportion or abundance of species at each station are also very clear. The diversity of species and their kinship can be observed in the following phylogenetic reconstruction.

**FIGURE 3:**
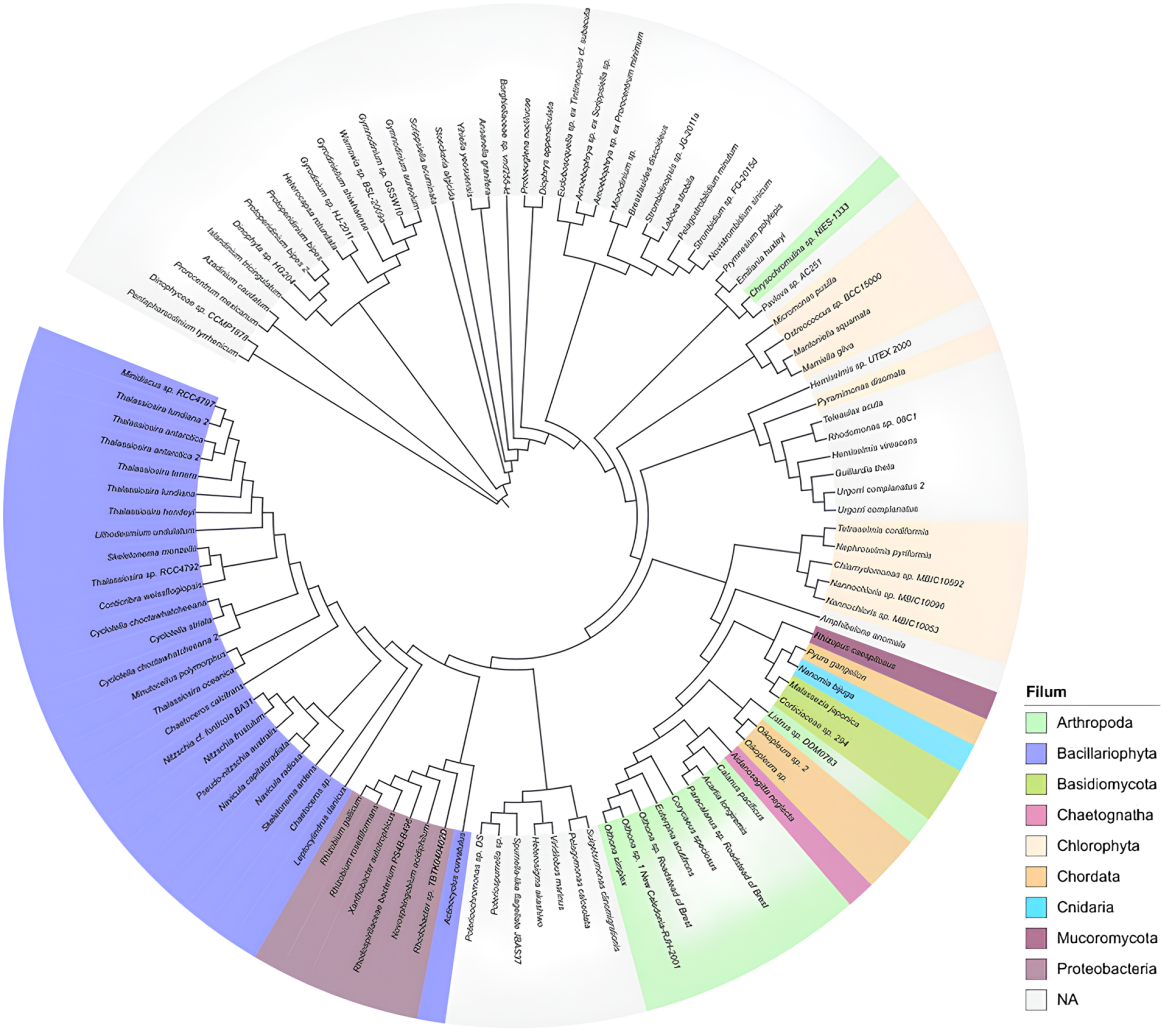
Phylogenetic identification results of aquatic organisms in the Lantebung Mangrove Forest on the basis of the 18S rRNA sequence.

**FIGURE 4:**
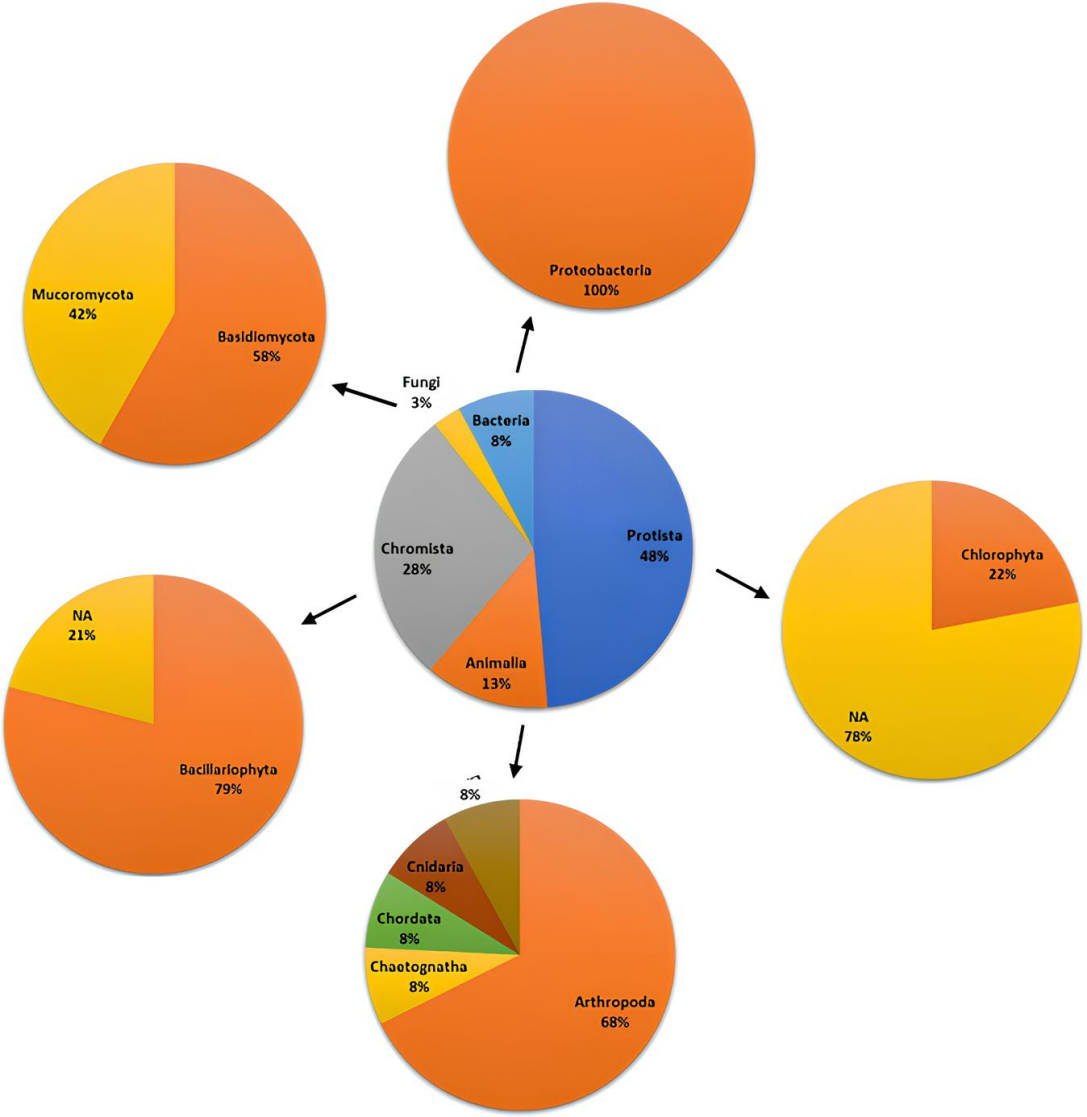
Pie chart of the percentage of species diversity at the kingdom and phylum levels of the Lantebung Mangrove Forest based on the 18S rRNA sequence.

Figure 3 shows that phylogenetic reconstruction helps to understand how current species and other groups of organisms are evolutionarily related. From here, we can determine the lineage or kinship relationships between these species. The data in Figure 4 show that the Makassar Lantebung mangrove is inhabited by species in the phylum Bacillariophyta, whereas species in the phyla Chaetognatha, Cnidaria, and Mucoromycota are the least common. In addition, the phylogenetic data above illustrate that the more or less branching of a species, the closer the kinship. Furthermore, the species that do not have much branching constitute the majority of the species that do not yet have a phylum, so the kinship relationship is not yet known due to the lack of appropriate data in the available database. The results of the analysis revealed that, in the Lantebung Mangrove Forest, 5 kingdoms have been identified. The five kingdoms are Fungi, Bacteria, Animalia, Protista, and Chromista. The kingdom Fungi was the kingdom of the least common species found in the sample (2.91%), whereas the most common species found was the kingdom Protista (48.54%) (Figure 4):

Figure 4 shows that the kingdom protista has the largest number of species (48.54%), which consists of 1 phylum, namely, 1 phylum; then, in the kingdom chromista, 28%, which consists of 1 phylum; then, there are as many as 12.62% animalia, which includes 4 phyla; Kingdom bacteria, as many as 7; 77%, which consists of 1 phylum; and the last, which consists of 2 phyla. Thus, the kingdom that has many types of phyla is kingdom animalia, as many as 4 phyla and kingdoms that have few types of phyla are protists, chromist, and bacteria.

**FIGURE 5:**
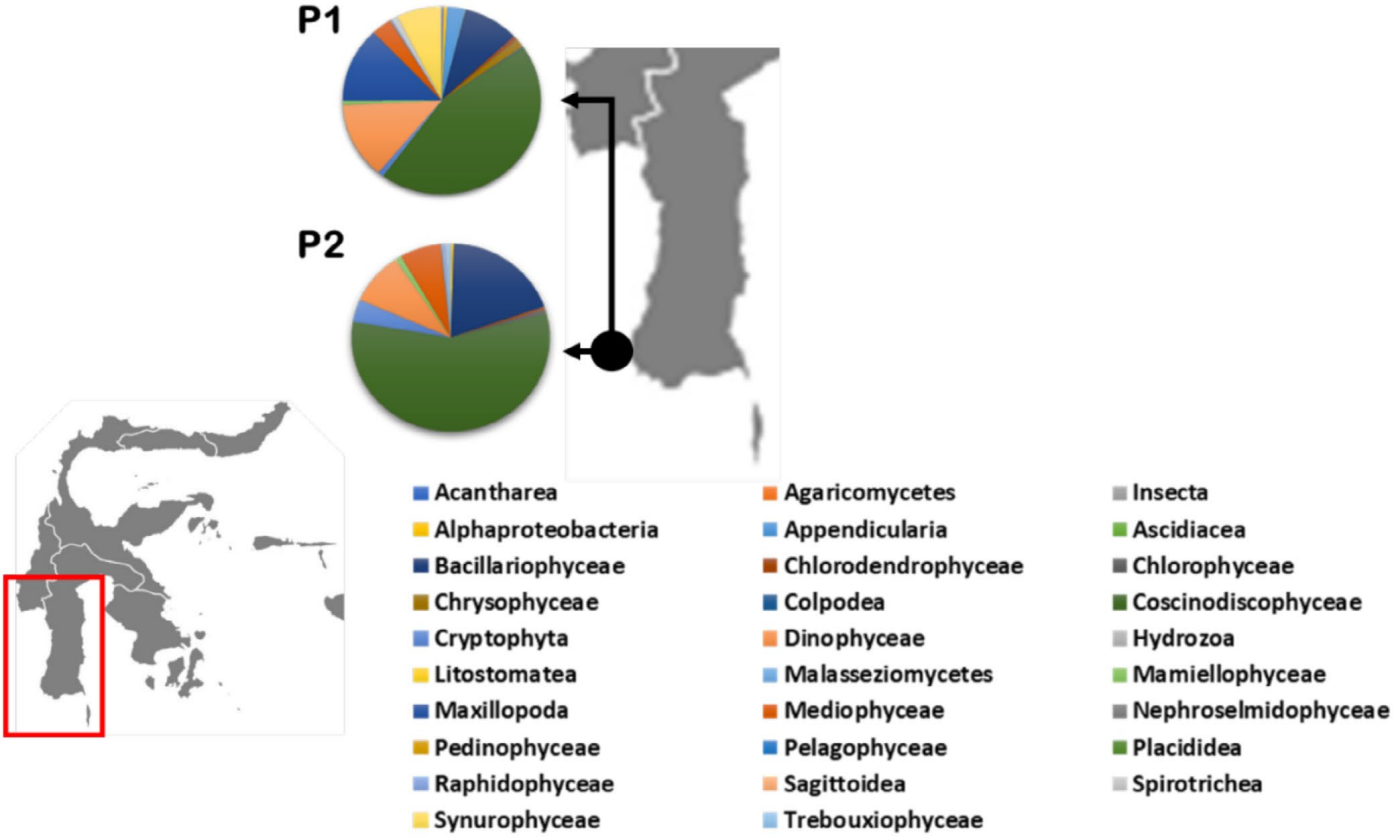
Pie chart of the percentage of Lantebung mangrove species at the class level at stations P1 and P2 on the basis of 18S rRNA gene sequences.

Figure 5 shows the sample analysis at station P1, which revealed 4 kingdoms, with chromista as the largest kingdom, namely, 56%, 30%, and 13% and the lowest kingdom was bacteria, accounting for as much as 1%. At station P1, there was high species diversity, with species in the Coscinodiscophyceae class having the highest relative diversity of 44% and those in the Hydrozoa class having the lowest relative diversity of only 0.05%.

The results of sample analysis at station P2 revealed that 5 kingdoms, including 82% of the Chromista kingdom, 17% of the protist kingdom, 1% of the bacteria, 0.30% of the fungi, and 0.11% of the animalia, were present. In this sample, the species with the highest relative diversity in the Coscinodiscophyceae class was 55.72%, and the species in the Placididea class had the lowest relative diversity of 0.02%.

**FIGURE 6:**
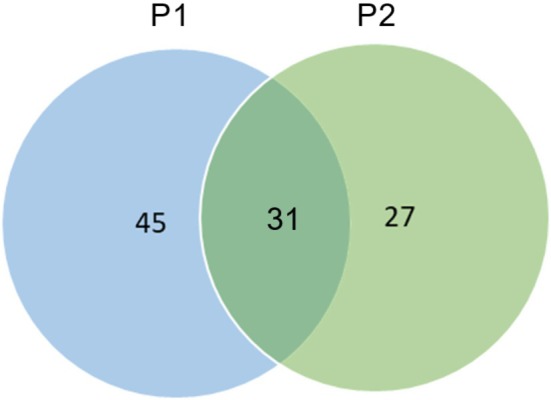
Venn diagram of the microbial species distribution in the Makassar Lantebung mangrove.

The above data indicate that species at the Coscinodinoscophyceae class level are the most dominant species between the two stations because they have the highest percentage of species at both stations, whereas species in the Sagittodea class are the class with the least number of species inhabiting both stations. The species relationships between the two sample stations are displayed in Figure 6.

Alpha‐diversity analysis showed that Station P1 exhibited higher microbial diversity than Station P2, reflected by higher Observed richness (76 vs. 56), Shannon index (4.31 vs. 3.99), and Simpson index (0.986 vs. 0.981) as depicted in Table 2. These results indicate that P1 supports a richer and more even microbial community structure, likely influenced by greater light penetration, stronger water exchange, and lower canopy shading compared with P2.

**TABLE 2:** Alpha‐diversity indices (Observed richness, Shannon, and Simpson) of microbial communities at stations P1 and P2.

Station	Observed richness	Shannon index	Simpson index
P1	76	4.31	0.98
P2	56	3.99	0.98

### 3.3 Abudance Microbial Potential

Overall, the number of species in sample P1 was 76, and that in sample P2 was as high as 58, which indicates that sample P1, which is outside the mangrove shade, has a greater diversity of microbial species than does sample P2, which is located in the shade of mangroves. Species that can be found in both samples, such as *Amoebophrya* sp. ex, *Scrippsiella* sp., *Borghiellaceae* sp. vnd255‐kt, *Chaetoceros calcitrans*, *Chaetoceros* sp., *Chlamydomonas* sp. MBIC10592, *Cyclotella choctawhatcheeana*, and *Cyclotella striata* , are a group of algae and protists that can be found in the same location for several common reasons, such as similar environments (e.g., salinity, temperature, light, and nutrients), ecological interactions (some species have complex ecologies, such as predator–prey relationships or mutualistic relationships). For example, *Amoebophrya* sp. is an endosymbiont organism living inside *Tintinnopsis cf. subacuta*. These interactions can affect the distribution and presence of these species at the same location, transport and displacement (microscopic organisms can easily be carried away by water flow, wind, or the transport of other organisms, such as zooplankton). This can lead to the dispersal of the same species to different locations and resource availability (the same location may provide the resources required by the species, such as nutrients or similar food sources). This can attract species that have similar resource requirements to congregate in the same location.

Furthermore, species limitations, such as better water circulation and open mangrove waters, tend to result in better water circulation in both samples because of the influence of tidal currents and connections with the ocean. This can provide a better supply of nutrients and influence the presence of microbes that depend on these resources. Open mangrove waters receive more sunlight than areas under the mangrove forest canopy. This can affect the photosynthetic activity of photosynthetic microbes, such as algae and phytoplankton. Open mangrove waters are often connected to larger aquatic ecosystems, such as estuaries or the open ocean. Interactions with the organisms and resources present in these ecosystems can affect the presence and diversity of microbes (Singh and Prabha 2019), which is why P1 has a greater abundance of species than P2 does.

### 3.4 Relative Read Abundance

In addition to determining the abundance of species, the eDNA metabarcoding analysis method can also detect the number of individuals in each species, where one sequence reading is equal to the reading of one individual.

The results of eDNA metabarcoding analysis revealed that there is a diversity of species in the Lantebung Mangrove Forest. The author identified 103 species belonging to 65 families, with a species abundance of 13,612 individuals on the basis of read sequence readings, where one read DNA sequence is assumed to represent one individual (Johnson et al. 2019).

**FIGURE 7:**
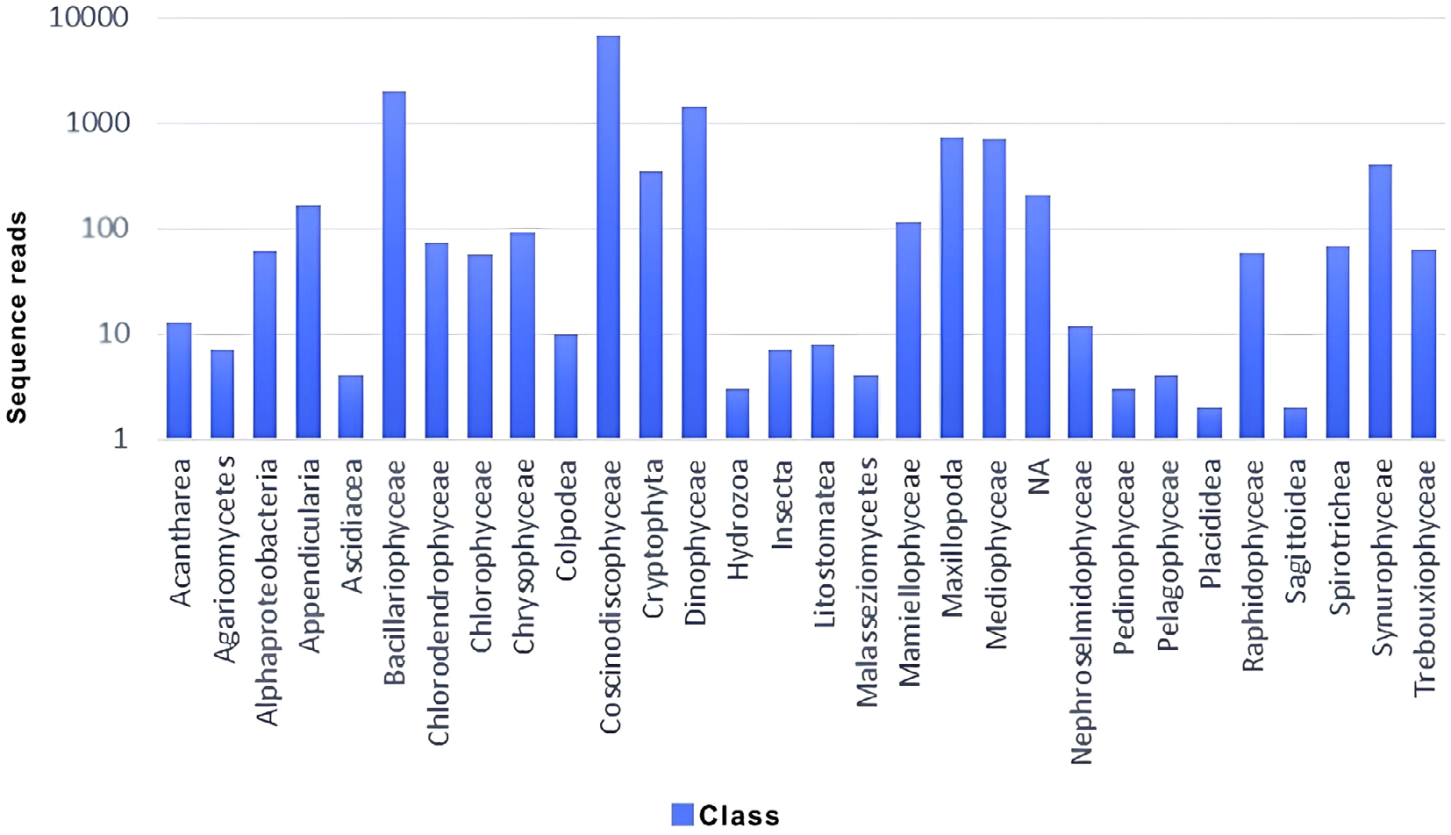
Abundance of individuals belonging to 29 classes on the basis of sequence reads.

Figure 7 depicts the abundance of individuals at the class level and clearly shows that the species in the class Coscnodisicophyceae is the class with the most sepsis, namely, 6926 individuals according to sequence readings. The lowest abundance of individuals is in members of the sagittodea class, which has only two individuals according to sequence readings. There are several unidentified individuals symbolized by the NA symbol at each level. At the phylum level, 2485 individuals do not have a phylum, 208 individuals are at the class level, 571 individuals are at the order level, 531 individuals are at the family level, and 114 individuals are at the genus level. The presence of individuals who cannot be specifically identified is due to the use of primers that are not appropriate or specific to incomplete databases. The identification results revealed that five kingdoms were present in the sample, namely, Bacteria, Protista, Chromista, Fungi and Animalia. Furthermore, the identification results are classified on the basis of each Kingdom.

#### 3.4.1 Kingdom Bacteria

Within the kingdom Bacteria, four genera and six species were identified in the mangrove water samples. Information on these types of bacteria can provide an overview of the conditions of mangrove waters, especially with respect to water quality and the presence of pathogenic microorganisms that can affect human and wildlife health. The following is a description of some of the bacteria found:

-
*Novosphingobium acidiphilum* : This genus of bacteria belongs to the phylum Proteobacteria and is found in many environments, including water and soil. Some Novosphingobium species are known to live by utilizing certain organic compounds, such as benzene, naphthalene, and phenol, which may be present in Lantebung mangrove water samples (Chaudhary and Khillare 2017)
-
*Rhizobium gallicum* and *Rhizobium rosettiformans* : These bacteria belong to the phylum Proteobacteria and are symbiotic bacteria that live in a mutualistic relationship with leguminous plants, assisting in nitrogen fixation. It is likely that leguminous plants live around the mangrove waters of Lantebung and provide favorable environments for these two bacterial species (Chaudhary and Khillare 2017).
- *Rhodobacter* sp. TBTK040402D and *Rhodospirillaceae* bacterium PS4B‐ B496: This genus of bacteria belongs to the phylum Proteobacteria and is found in various environments, including water. Some Rhodobacter and Rhodospirillaceae species are known to be photosynthetic bacteria capable of producing energy from sunlight through the process of photosynthesis (Chaudhary and Khillare 2017). The presence of these bacterial species is related to the presence of sunlight sources in Lantebung mangrove waters.
-
*Xanthobacter autotrophicus* : This genus of bacteria belongs to the phylum Proteobacteria and is found in a variety of environments, including soil and water. Some *Xanthobacter* species are known to live by utilizing inorganic compounds, such as ammonia and nitrogen oxides, as sources of energy and nutrients (Chaudhary and Khillare 2017). The presence of this bacterial species is related to the presence of these inorganic compounds in Lantebung mangrove waters.

Lantebung mangrove waters are complex environments rich in natural resources. The presence of various bacterial species is related to different environmental conditions, such as the presence of leguminous plants, sunlight sources, and certain organic and inorganic compounds. Metabarcoding identification can provide useful information for understanding the diversity and ecosystem there.

#### 3.4.2 Kingdom Protista

In the Kingdom Protista, 37 species were identified in mangrove water samples. Protists are a group of organisms that play important roles in aquatic ecosystems, especially in the food chain. Protist identification can provide an overview of the phytoplankton and planktonic composition of mangrove waters (Di Cesare et al. 2018).

The results revealed the presence of a variety of protists, including algae, protozoa, and other single‐celled organisms. Some of the algae organisms identified included *Tetraselmis cordiformis* and *Pseudonitzschia australis* . Algae play an important role in mangrove ecosystems as primary producers and provide food sources for other organisms (Di Cesare et al. 2018). In addition, protozoa such as *Protoeuglena noctilucae*, *Gyrodiniellum shiwhaense*, and *Strombidium* spp. are heterotrophic organisms that can act as predators, decomposers, and as part of the food chain in aquatic ecosystems. Furthermore, several types of dinoflagellates, Dinophyceae sp. CCMP1878 and Gymnodinium aureolum, which can cause red algal blooms that can affect the surrounding environment, were also detected. Dinoflagellates are known for their ability to produce a toxin called saxitoxin, which can cause severe poisoning in humans and marine animals. The presence of dinoflagellates in seawater can cause a phenomenon known as a “red tide” or “bloom,” where seawater appears reddish in color owing to the overgrowth of these dinoflagellates (Di Cesare et al. 2018).

In this study, the presence of organisms such as *Pelagostrobilidium minutum* and *Euduboscquella* sp. *ex Tintinnopsis cf. subacuta* suggests a link between the mangrove aquatic ecosystem and the wider marine environment. These organisms can be considered important indicators for understanding water dynamics and nutrient transfer processes between mangrove and marine ecosystems (Di Cesare et al. 2018).

Nannochloris sp. MBIC10053 and Nannochloris sp. MBIC10096 were found. *Nannochloris* is a microalga that usually lives in freshwater and sea environments. This alga has a small cell shape and is generally spherical. *Nannochloris* sp. acts as a primary producer in aquatic ecosystems. *Amoebophrya* sp. ex *Prorocentrum minimum* and *Amoebophrya* sp. ex *Scrippsiella* sp. Amoebophrya are parasites that live inside the cells of other protists, including diatoms and dinoflagellates. Several species of Amoebophrya were found to parasitize *Prorocentrum minimum* and *Scrippsiella* sp., infecting host cells and affecting their growth. The presence of Amoebophrya can affect the abundance and diversity of protists in water.

The diversity of protists found in this study also reflects a healthy and well‐functioning mangrove aquatic environment. Protists play a role in various ecosystem functions, including as primary producers, as part of the food chain, and as decomposers of decomposed organic matter. The presence of diverse and balanced protists can indicate ecosystem stability and good water quality (Di Cesare et al. 2018).

#### 3.4.3 Kingdom Chromista

Kingdom Chromista is a group of unicellular or multicellular eukaryotic organisms that have combined characteristics of plants, animals, and fungi. Within this group are various types of phytoplankton that play important roles in maintaining the balance of the marine ecosystem (Andersen 2004). Some of them have distinctive characteristics that can be used as markers of changing environments and ocean conditions (Hoppenrath et al. 2009; Guiry and Guiry 2021). The following is about phytoplankton, and some discussion about the identification results. The shape of *Actinocyclus curvatulus* is flat and round and looks like a gear when viewed from above. This diatom has strong cell walls and a polar or pinnacle shape that rises upward.

*Chaetoceros calcitrans* and *Chaetoceros* sp. are a type of diatom that has a tube‐like or cylindrical shape with a grooved cell wall. Both diatoms play important roles in the carbon and nitrogen cycles in marine waters. *Cyclotella choctawhatcheeana* and *Cyclotella striata* have a flat, round shape and look like gears when viewed from above. These diatoms are often used as indicators of water cleanliness and environmental quality. *Thalassiosira antarctica* , *Thalassiosira hendeyi*, *Thalassiosira lundiana*, *Thalassiosira oceanica*, and *Thalassiosira* sp. RCC4792 are types of diatoms. The shape is round or oval with tubular cell walls. *Nitzschia cf. fonticola BA31* and *Nitzschia frustulum* are diatoms that can be found in freshwater and marine waters. The shape is flat and long with thin cell walls. *Nitzschia* is often used as an indicator of water cleanliness and environmental quality. *Skeletonema ardens* and *Skeletonema menzellii* are diatoms that can be found in marine and estuary waters. It has a stick‐like shape with smooth cell walls.

*Guillardia theta* is a microalga that belongs to the cryptophyte group. This alga has an oval or round cell shape and is commonly found in marine waters. *Hemiselmis* sp. *UTEX 2000* and *Hemiselmis virescens* have a round or oval cell shape. *Hemiselmis* sp. and *Hemiselmis virescens* play a role in the food chain and oxygen production in water. *Prymnesium polylepis* is a type of dinoflagellate that can cause toxic algal blooms. When overgrown, *Prymnesium polylepis* produces toxins that can kill fish and other marine life, causing economic and ecological losses.

#### 3.4.4 Kingdom Fungi

Among the kingdom fungi, three genera and three species were identified in the mangrove water samples. Fungi are generally not found in many waters, but their presence can have a significant effect on water quality and mangrove aquatic ecosystems.

*Rhizopus caespitosus* is a type of fungus that belongs to the Zygomycetes group. This fungus is found on a variety of organic substrates and is sometimes involved in the decomposition of organic matter. Some Rhizopus species can cause infections in humans, especially in individuals with weakened immune systems (Nilsson et al. 2019).

*Malassezia japonica* is a common fungus found on the skin of humans and some animals. Some Malassezia species can cause infections in humans, especially in individuals who have certain health problems, such as sensitive skin or a weakened immune system (Nilsson et al. 2019).

*Corticiaceae* sp. 294 is a type of fungus that belongs to the Basidiomycetes group. This fungus has an important role in the decomposition of organic matter in the environment. Corticiaceae spp. are often found on dead wood substrates and can help in the process of wood decomposition (Nilsson et al. 2019).

In the context of metabarcoding identification, the results of fungal species identification provide important information about the composition and structure of the fungal community in the sampled environment. The presence of these species can provide clues about the ecological function of the environment, such as the presence of available organic substrates or the presence of potential hosts for parasitism by certain fungi. In addition, this information can provide greater insight into the interactions between fungi and other organisms in the environment, including humans, if the species has pathogenic potential (Nilsson et al. 2019).

#### 3.4.5 Kingdom Animalia

Within the Kingdom of Animalia, 13 species were identified in the mangrove water samples. Faunal identification can provide information on the diversity of wildlife found in mangrove waters and provide information on the conservation status and sustainability of mangrove ecosystems. Some examples of species identified are Acartia iongiremis, *Calanus pacificus* , *Corycaeus speciosus* , and Oikopleura sp., which are types of zooplankton that act as food for fish and other marine animals. In addition, some species, such as *Nanomia bijuga* , are jellyfish or sea jelly (Mahe et al. 2017). Overall, the results of identification by metabarcoding techniques in mangrove water samples revealed diverse organisms from various kingdoms.

## 4 Conclusion

This study successfully identified 103 OTUs consisting of five kingdoms, namely, Protista 50 OTUs (48.54%), Chromista 29 OTUs (28%), Animalia 13 OTUs (12.62%), Bacteria 8 OTUs (7.77%), and Fungi 3 OTUs (2.91%). In addition, 290 OTUs (73.79%) were not classified at the taxonomic level because the OTUs were not yet available in the database. In the P1 sample, 76 OTUs (73.78%) presented greater species diversity than did the P2 sample, with 58 OTUs (54.36%), but in the P2 sample, 7906 individuals (58.08%) presented greater relative abundance than did the P1 sample, with 5706 individuals (41.91%). This study serves as an important first step in characterizing the microbial community structure in the Lantebung mangrove by metabarcoding 18S rRNA. Despite the limited sample size and environmental parameters, this research provides a valuable baseline for future biodiversity assessments and monitoring efforts in Indonesian mangrove ecosystems.

## Author Contributions

**Siti Halimah Larekeng:** conceptualization (lead), supervision (equal), writing – original draft (equal). **Mohammad Basyuni:** data curation (equal), formal analysis (equal), supervision (equal), writing – original draft (equal), writing – review and editing (equal). **Andi Aznan Aznawi:** software (equal), writing – original draft (equal). **Irmawati Irmawati:** methodology (equal), writing – original draft (equal). **Iswanto Iswanto:** visualization (equal), writing – original draft (equal). **Muhammad Saldy:** writing – original draft (equal). **Alfian Mubaraq:** writing – original draft (equal). **Bejo Slamet:** writing – original draft (equal). **Elham Sumarga:** formal analysis (equal), investigation (equal), writing – review and editing (equal). **Virni Budi Arifanti:** writing – review and editing (equal). **Hayssam M. Ali:** writing – review and editing (equal).

## Funding

This work was supported by the Indonesian Research Collaboration (Riset Kolaborasi Indonesia) between USU‐UNHAS and ITB (No. 01369/UN4.22/PT.01.03/2024). Partly was supported by e‐ASIA Joint Program from Endowment Fund for Education Agency (Lembaga Pengelola Dana Pendidikan), Ministry of Finance, Republic of Indonesia year 2024–2025 (No. 019/E5/PG.02.00/PRPB BATCH 2/2024).

## Ethics Statement

The authors have nothing to report.

## Conflicts of Interest

The authors declare no conflicts of interest.

## Data Availability

The data supporting the findings of this study are publicly available to ensure the transparency and reproducibility of the research. The dataset employed in generating graphs and tables, which form the basis of this study, is accessible at https://doi.org/10.6084/m9.figshare.29421434.
